# Successful Treatment of Rapidly Evolving Cutaneous Leishmaniasis With Amphotericin B and Miltefosine in an Immigrant From Venezuela

**DOI:** 10.1093/ofid/ofad683

**Published:** 2023-12-28

**Authors:** Danielle M Mullis, Evan Shegog, Lucy Studemeister, Michael Hwang

**Affiliations:** Department of Cardiothoracic Surgery, Stanford University School of Medicine, Stanford, California, USA; Department of Internal Medicine, Santa Clara Valley Medical Center, San Jose, California, USA; Department of Internal Medicine, Santa Clara Valley Medical Center, San Jose, California, USA; Department of Internal Medicine, Santa Clara Valley Medical Center, San Jose, California, USA

**Keywords:** leishmaniasis, *Leishmaniasis braziliensis*, miltefosine

## Abstract

Leishmaniasis is a vector-borne disease uncommonly encountered in the United States. This case report describes a 54-year-old man presenting with rapidly progressing, pruritic, painful ulcerative lesions after recently immigrating from Venezuela. A punch biopsy confirmed infection with *Leishmaniasis braziliensis*. He was successfully treated with amphotericin B and miltefosine.

Leishmaniasis is a neglected tropical disease caused by the protozoan parasites of the genus *Leishmania* and transmitted by a sandfly bite [1–3]. Leishmaniasis presents with 3 distinct clinical syndromes, consisting of visceral leishmaniasis (VL), cutaneous leishmaniasis (CL), and mucosal leishmaniasis (ML) [4]. CL is further divided into the geographic distribution of CL, specifically Old World CL and New World CL, referring to the Eastern Hemisphere and Western Hemisphere, respectively [1].

New World CL is endemic throughout Central and South America [1, 3]. The primary reservoirs are forest rodents, and the vectors are the *Lutzomyia* species of sandflies [1]. Disease is common among those residing in rural regions or those who work in the forest edge, such as military personnel or construction workers [1].

Leishmaniasis is not endemic to the United States and not a disease that is mandatory to report, except in Texas [5]. Therefore, it is difficult to assess the magnitude of infection. Given increased immigration from Central and South America to the United States, as well as limited availability of the oral medication to treat this infection, it is of utmost importance that providers can identify leishmaniasis in a timely manner [6].

We present the case of a previously healthy 54-year-old man who was identified as having a rapidly evolving case of CL, and was treated with intravenous (IV) amphotericin B and oral miltefosine. This case report identifies the risk factors, classic disease presentation, diagnosis, and treatment modalities for New World CL caused by *Leishmania braziliensis.* Although this patient's clinical presentation of leishmaniasis was rapidly evolving, he demonstrated an excellent response to treatment. This case also highlights various barriers that immigrants may face when seeking healthcare. The primary objective is to guide practitioners to stay vigilant for this diagnosis, especially given the significant increases in immigration from Central and South America to the United States [6].

## CASE REPORT

A previously healthy 54-year-old man presented to a California emergency department (ED) with 2 ulcerative skin lesions on his left wrist and distal left forearm. The patient reported traveling from Venezuela to California via foot, bus, and train 4 weeks prior to presentation. His path traversed Colombia, Panama, Costa Rica, Nicaragua, Honduras, Guatemala, and Mexico, including through the Darien gap. He believes he was bitten by a bug while sleeping outside in the jungles of Panama. Initially, the lesions were pruritic and erythematous, but they gradually developed into large, painful ulcerations. He was initially diagnosed with cellulitis upon first presentation to the ED and discharged on a 10-day course of doxycycline.

**Figure 1.**
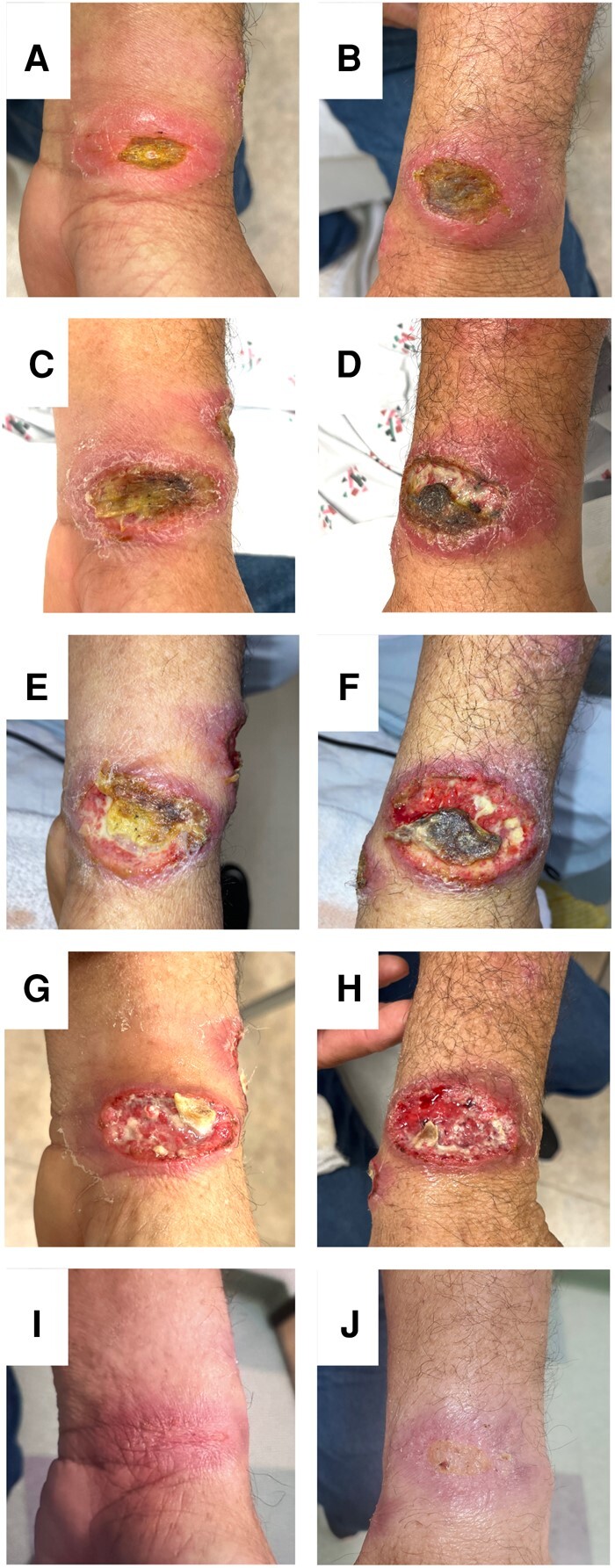
Cutaneous Leishmaniasis Lesions on the Left Wrist and Forearm. *A*, Lesion with an erythematous border on the left wrist approximately 12 days preadmission. *B*, Ulcerative lesion with an erythematous border on the distal left forearm 12 days preadmission. *C*, The wrist lesion on the day of admission (approximately 5 cm in diameter); intravenous amphotericin B (AmB) was started. *D*, The forearm lesion on the day of admission (approximately 5 cm in diameter). *E*, The wrist lesion on the day of discharge (4 days since admission and starting AmB; the patient was discharged on miltefosine). *F*, The forearm lesion on the day of discharge (4 days since admission and starting AmB; the patient was discharged on miltefosine). *G*, The wrist lesion 4 days after starting miltefosine. *H*, The forearm lesion 4 days after starting miltefosine. *I*, The wrist lesion approximately 1 week after finishing the 28-day course of miltefosine. *J*, The forearm lesion approximately 1 week after finishing the 28-day course of miltefosine.

The patient returned to the ED 5 days later due to worsening necrosis, pain, and pruritis of his wounds (Figure 1*A* and 1*B*). He was incidentally found to have subcentimeter lymph nodes in the upper arm proximal to the 2 large lesions. The infectious disease team was consulted, who recommended a punch biopsy of the wound to confirm their suspicion of leishmaniasis. Paraffin-embedded tissue was sent out to a local academic hospital for further genetic workup. He was discharged and advised to follow up on an outpatient basis once the punch biopsy results returned.

After 12 days, the patient returned to the ED for significantly worsening pain, pruritus, and swelling of the 2 large lesions (Figure 1*C* and 1*D*). He was also found to have significant axillary lymphadenopathy as well as additional painful erythematous lesions on his right cheek and bilateral arms. At this time, results from punch biopsy were completed and confirmed the diagnosis of leishmaniasis. Histopathology noted intracytoplasmic small round hematoxylinophilic formations suggestive of *Leishmania* amastigotes. DNA sequencing was performed on the paraffin-embedded tissue sent to a local academic hospital, which identified *Leishmania (Viannia) braziliensis*. The test was developed by the academic hospital and its performance characteristics were determined by the hospital's laboratory; the sensitivity of the test is 96.4% and specificity of the test is 98.3% using culture as the reference method.

Given his subjective fevers, new satellite lesions, and worsening ulcerative, painful, pruritic wounds, the patient was directly admitted for initiation of 3 mg/kg/day intravenous liposomal amphotericin B. The patient stayed in the hospital for 4 days receiving liposomal amphotericin while waiting for miltefosine to be approved and delivered to the hospital (Figure 1*E* and 1*F*). The patient was discharged on 50 mg miltefosine tablets to be taken by mouth 3 times per day for 28 days. Four days after starting miltefosine, the lesions already began to show marked improvement (Figure 1*G* and 1*H*), and 1 week after finishing the 28-day course, the lesions were almost completely healed. As of 5 months after discharge from the hospital, the patient was healthy, had not been rehospitalized, and reported no disease relapse.

## DISCUSSION

While there are 3 distinct clinical syndromes (VL, CL, and ML), the clinical manifestations of leishmaniasis can vary with the patient's immune response [7–9]. Diffuse CL results when the patient has a poor T-cell response but a strong macrophage response to *Leishmania* parasites, and it typically presents with multiple nonulcerative nodules [10]. Disseminated leishmaniasis, commonly found in the New World, is characterized by a poor macrophage response to *Leishmania* parasites and typically presents with many lesions distributed over multiple sites of the body [1, 10]. *Leishmania braziliensis* is 1 of the 2 main subgenera identified as a major cause of disseminated infections [8]. Disseminated infections are difficult to treat, which makes early identification and treatment extremely important [11].

This patient's experience before hospitalization demonstrates how challenging it can be to identify CL early on in its disease course. As demonstrated in this case, the patient was first diagnosed with cellulitis when he presented to the ED with 2 erythematous lesions. A thorough history and close patient follow-up can aid in making a correct diagnosis. Patients who are military personnel, travelers, or immigrants from endemic regions with leishmaniasis presenting with unusual cutaneous ulcers should prompt physicians to obtain a skin biopsy sample as early as possible, as genetic and histologic analyses of the tissue are crucial to confirm the diagnosis. In our patient's case, he was rapidly developing new lesions and lymphadenopathy. Also, his lesions were growing significantly more painful, which is unusual for leishmaniasis. While superimposed bacterial infection was initially suspected due to the painful and erythematous nature of the lesions, aerobic cultures demonstrated no growth, and anaerobic culture demonstrated only rare growth of *Propionibacterium acnes*.

By the time the patient was admitted to the hospital, the biopsy results were back, and the diagnosis could be confirmed. The patient was otherwise healthy and stable; therefore, he was discharged on oral miltefosine instead of remaining in the hospital for amphotericin B. Importantly, although incompletion of the 14-day amphotericin B regimen could possibly induce drug resistance, it was felt the risks of staying in the hospital receiving IV medication (ie, nosocomial infections and blood-borne infections) outweighed the benefits of finishing the 14-day IV amphotericin B course, and he was, therefore, discharged on a 28-day course of oral miltefosine.

Successful identification of the subgenus of *Leishmania* species is necessary for choosing the correct treatment. However, skin biopsies requiring histopathological staining and genetic analysis can take several days to result, thus leading to delays in appropriate treatment. Even with early identification, it may still take several days to obtain the proper treatment—in this case miltefosine—due to requirements in authorization from the pharmacy, hospital, and insurer.

According to the Centers for Disease Control and Prevention (CDC), the treatment of leishmaniasis depends on both host and parasite factors. Specifically, some treatments are only effective against specific *Leishmania* species and can also depend on the geographic region [12]. Liposomal amphotericin B is approved by the US Food and Drug Administration (FDA) to treat VL, but it requires extended hospitalization and data utilizing this treatment are still somewhat limited [13]. The FDA has approved miltefosine for the treatment of CL, ML, and VL caused by specific *Leishmania* species, one of which is *L braziliensis,* thus further demonstrating the importance of obtaining an early biopsy to guide treatment [12, 14]. Of note, other countries with endemic leishmaniasis have alternative approved therapeutic agents, such as Glucantime, which are not recommended by the CDC nor approved by the FDA [15].

In our patient's case, the 2 large lesions and satellite lesions demonstrated significant improvement only days after starting miltefosine (Figure 1*G* and 1*H*) and outstanding improvement by 1 week after finishing the 28-day course of miltefosine (50 mg, 3 times per day) (Figure 1*I* and 1*J*). The patient reported that by 5 months after discharge, his lesions were completely healed, thus providing hope that despite his initial worsening leishmaniasis, his treatment has been extremely fast and successful.

## Notes

***Patient consent.*** The patient's informed written consent was obtained. All details and photographs have been anonymized as much as possible. This case report adheres to the standards currently applied in California.
